# Steam-Processed *Stauntonia hexaphylla* (Thunb.) Decne Fruit Stimulates Osteoblast Differentiation in MC3T3-E1 Cells and Inhibits Osteoclastogenesis in RAW 264.7 Cells

**DOI:** 10.3390/ijms26178411

**Published:** 2025-08-29

**Authors:** Muhammad Awais, Reshmi Akter, Md Niaj Morshed, Jong Hak Kim, Byoung Man Kong, Dong Wook Lee, Sung Keun Choi, Chang Soon Lee, Jong Chan Ahn, Deok Chun Yang, Jong Min Lee

**Affiliations:** 1 Department of Biotechnology, College of Fisheries Sciences, Pukyong National University, Busan 48547, South Gyeongsang, Republic of Korea; muhammadawais@pknu.ac.kr; 2 Graduate School of Biotechnology, College of Life Sciences, Kyung Hee University, Yongin-si 17104, Gyeonggi-do, Republic of Korea; reshmiakterbph57@gmail.com (R.A.); niajmorshed96@khu.ac.kr (M.N.M.); kong2167@naver.com (B.M.K.); 3 Hanbangbio Inc., Yongin-si 16954, Gyeonggi-do, Republic of Korea; jhkim@rgk.co.kr (J.H.K.); dwlee5001@rgk.or.kr (D.W.L.); jongchanahn7@khu.ac.kr (J.C.A.); 4 Daedong Korea Ginseng Co., Ltd., 86, Gunbuk-ro, Gunbuk-myeon, Geumsan-gun 32718, Chungcheongnam-do, Republic of Korea; ddgc0815@ddkorea.co.kr (S.K.C.); hippo8270@ddkorea.co.kr (C.S.L.); 5 Department of Oriental Medicinal Biotechnology, College of Life Sciences, Kyung Hee University, Yongin-si 17104, Gyeonggi-do, Republic of Korea

**Keywords:** osteoporosis, *Stauntonia hexaphylla*, steaming, osteoclastogenesis, osteoblastogenesis

## Abstract

*Stauntonia hexaphylla* (Thunb.) Decne (SH), a medicinal plant from the Lardizabalaceae family, holds traditional importance in East Asia for treating rheumatism. Steam treatment is commonly applied to enhance its medicinal properties, but the chemical and biological changes resulting from this process remain unexplored. This study compared steamed and untreated SH fruit (SHF) extracts, analyzing their chemical composition, antioxidant activity, and effects on bone health using in vitro models. Steamed SHF extracts exhibited increased levels of 5-hydroxymethylfurfural (5-HMF), total flavonoids, phenolics, and enhanced antioxidant activity. Bone health assessment using osteoclasts differentiated from RAW 264.7 cells and osteoblasts from MC3T3-E1 cells revealed that steamed extracts promoted alkaline phosphatase activity, calcium nodule formation, and collagen synthesis in osteoblasts while inhibiting tartrate-resistant acid phosphatase (TRAP) activity in osteoclasts. Additionally, steamed SHF extracts effectively modulated gene expression related to osteoclastogenesis and osteoblastogenesis by downregulating TRAP, NFTAc1, RANK, MMP9, c-Fos, and TRAF6 while upregulating ALP, Runx2, BGLAP, Col1a1, and OPG. The component 5-HMF played a pivotal role in promoting alkaline phosphatase and inhibiting TRAP activities. These findings suggest that steamed SHF may offer a promising therapeutic approach for postmenopausal osteoporosis.

## 1. Introduction

Osteoporosis is characterized by a reduction in bone mass, decreased bone density, and degeneration of the microstructure of bone tissue, often due to a deficiency of calcium and proteins. This metabolic bone disease occurs when bone resorption outpaces bone formation, leading to an imbalance in bone remodeling. Osteoporosis is a substantial health concern that has become more prevalent recently, primarily due to the increasing aging population [1]. Various factors contribute to bone abnormalities, including inadequate calcium and protein intake, hormonal imbalances associated with menopause, obesity, and reduced physical activity [2]. Unhealthy lifestyle habits, such as the frequent consumption of processed foods and heavy smoking and drinking, can also contribute to joint and bone diseases [3]. Although bone resorption inhibitors such as bisphosphonates and denosumab were initially developed for osteoporosis treatment, individuals using them often encounter serious adverse effects [4]. Bisphosphonates may lead to esophageal or stomach inflammation, causing pain, heartburn, nausea, vomiting, or digestive issues [5].

Recent investigations have unveiled the positive effects of natural metabolites on bone formation and osteoclastogenesis [6]. Notably, certain flavonoid constituents including quercetin, rutin, and naringenin have been found to prevent bone resorption and stimulate bone formation [7]. Additionally, isoflavones derived from soybeans have been found to enhance the osteogenic activity of osteoblasts [8]. Oxidative stress has also been linked inversely to decreased bone density, promoting bone resorption through the activation of osteoclast differentiation [9]. These findings suggest that natural substances with antioxidant properties, such as flavonoids, may offer potential for osteoporosis prevention and treatment.

*Stauntonia hexaphylla* (Thunb.) Decne (SH) belongs to the Lardizabalaceae family and is a subtropical vine plant predominantly distributed in Korea, Japan, and China [10]. Various parts of SH, including its fruits, leaves, stems, and roots, have traditionally been used in oriental medicine for their analgesic, sedative, and diuretic attributes [11]. Recent research has reported SH’s potential to enhance post-menopausal osteoporosis recovery [12], exhibit anti-inflammatory properties [11], mitigate alcoholic liver damage [13], and alleviate fatigue [14]. The fruit of SH (SHF) is esteemed as a health food due to its rich content of vitamin C and vital minerals, such as calcium (Ca), potassium (K), and magnesium (Mg). It also contains various phytochemicals, such as flavonoids, polyphenols, and phenolic acids [15]. It has been reported that the bone formation-promoting and bone resorption-inhibiting activities of SH are caused by some flavonoids or phenolic acids existing in the leaves or fruits [16]. SH has recently gained prominence in the health food markets of Korea, Japan, and China, where it is known by names such as ‘Mulkul,’ ‘Mube,’ and ‘Ye-mu-gua’, respectively [11].

In the realm of food and natural product enhancement, a spectrum of techniques is employed, ranging from heating, drying, and microwave treatment to fermentation and enzyme treatment, aimed at bolstering their physiological attributes [17]. Among these methods, the steaming process is utilized to amplify the content and bio-accessibility of valuable phytochemicals, including phenolic metabolites, while preserving the inherent constituents of the raw materials. Traditional oriental medicine encompasses a practice referred to as ‘Bupjae,’ in which primary processed medicinal ingredients undergo reprocessing according to specific procedures to heighten their quality and effectiveness. Steaming processing is one of the methods traditionally associated with ‘Bupjae’ [18]. Phenolic metabolites in natural products, fruits, vegetables, and foods can be found in both free and bound forms. The application of steaming or heat treatment to these materials can result in an increase in bound phenolic metabolites and pseudo-melanin substances, leading to browning due to the oxidation reaction of o-diphenols [19]. Indeed, it has been demonstrated that steaming chestnuts leads to a significant increase in both bound and free phenolic metabolites [20]. However, the effects of steaming *S. hexaphylla* fruit (SHF) on dynamic modifications has not been performed yet.

Hence, our study was conducted with the expectation that dynamic modifications in phenolic metabolites and other bio-active substances would transpire when SHF is subjected to steaming processes. It is anticipated that these altered constituents will exert a positive impact on physiological activities linked to osteoporosis.

## 2. Results

### 2.1. The Effect of Steaming of SHF on the Properties and Extract Yield

The color of SHF and its extracts was changed to dark-brown by the steaming, and it was increased gradually with the number of steaming treatments (Figure S1). The texture of the fruits changed from a rough structure for unsteamed *S. hexaphylla* fruit (ST-C), one-time steamed *S. hexaphylla* fruit (ST-1), and two times steamed *S. hexaphylla* fruit (ST-2) to a fine powder for three times steamed *S. hexaphylla* fruit (ST-3). The yield of the extract of SHF as a solid part was increased to 15% in ST-2; however, it was lowered below the control level in ST-3. The yield of the SHF extract, measured as the solid content, increased by 15% in ST-2, reaching 506 mg/g compared to 440 mg/g in the untreated control. However, the yield decreased to 415 mg/g in ST-3, falling below the control level (Figure S1).

### 2.2. The Effect of Steaming of SHF on Phenolic Metabolite Content and Free Radical Scavenging Ability

**Figure 1:**
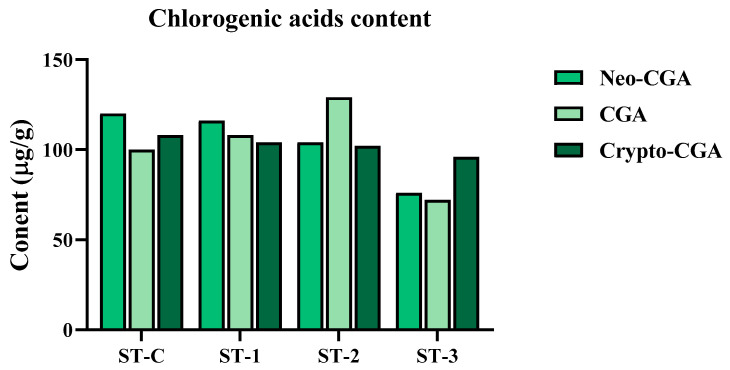
The effect of steaming SHF on the content of chlorogenic acids.

Figure 1 shows the content of CGAs including neo-CGA and crypto-CGA, which is one of the major secondary metabolites of SHF. The contents of neo-CGA, CGA, and crypto-CGA in unsteamed fruits (ST-C) were 120, 100, and 108 µg/g, respectively, and these values showed no changes until steaming for two times. However, when the fruits were steamed three times under the same conditions, the content of CGAs was greatly reduced. In all samples, the ratio of the three CGA isomers was unchanged.

The total phenolic content (TPC) of unsteamed ST-C was 18.4 ± 0.02 μg/mg extract, expressed as gallic acid equivalents (GAE). After one cycle of steaming (ST-1), the TPC increased by 61% to 29.7 ± 0.03 μg/mg. Following two cycles of steaming (ST-2), the increase reached 66%, while three cycles (ST-3) resulted in a 33% increase compared to the control (Figure S2A).

Similarly, the total flavonoid content (TFC) significantly increased from 1.46 ± 0.01 μg/mg extract in ST-C to 10.94 ± 0.02 μg/mg (747%) and 11.19 ± 0.02 μg/mg (766%) in ST-1 and ST-2, respectively. The TFC was expressed as micrograms of rutin equivalents (RE) per milligram of plant extract (μg RE/mg), a standard unit for quantifying the flavonoid concentration. However, after three cycles of steaming (ST-3), the TFC slightly decreased to 8.95 ± 0.01 μgRE/mg extract, representing a 613% increase relative to the control (Figure S2B).

As shown in Figure S2C, the untreated SHF extract was 607 μg GAE/g on a dry weight basis. When the SHF were steamed once, it increased by 59% to 965 μg GAE/g, and when it was steamed twice, it significantly increased by 84% to 1118 μg GAE/g. However, when steamed three times, the increase rate decreased slightly to 917 μg GAE/g.

### 2.3. The Effect of Steaming of SHF on Cell Proliferation in MC3T3-E1 Cells

We assessed cell proliferation using the MTT assay with SHF concentrations ranging from 12.5 to 200 μg/mL. Following a 22 h exposure to extract of the steamed SHF, a proliferative effect was observed, with a noticeable increase in cell proliferation up to a concentration of 125 µg/mL. However, this proliferative effect was not evident at higher concentrations. Subsequently, following a 48 h treatment period, it was observed that the proliferative effect of the steamed SH extracts was relatively diminished in comparison to the 24 h treatment, particularly at higher concentrations. Consequently, for subsequent experimental investigations, the concentration of up to 100 µg/mL of SH extracts was chosen. This decision was based on the observation that compared to the control group, it demonstrated a significant cellular proliferation effect. This concentration was deemed suitable for further experiments to study the effects of SH extracts on MC3T3-E1 cells (Figure S3A–C).

### 2.4. The Effect of Steaming of SHF on ALP Activity in MC3T3-E1 Cells

**Figure 2:**
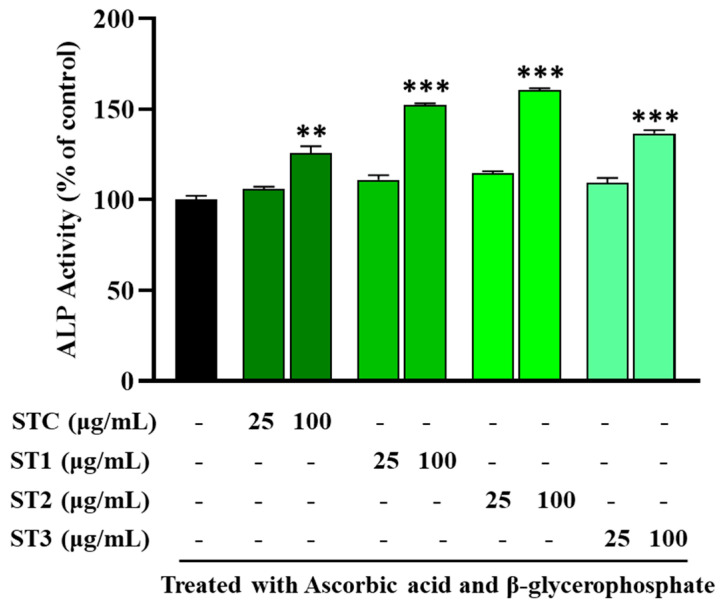
Evaluation of SHF impact on ALP (alkaline phosphatase) activity in MC3T3-E1 cells over a 7-day culture period with different SHF concentrations (25 to 100 μg/mL). The presented data represent the mean ± standard deviation from three independent experiments. Statistical analysis indicated significant differences, marked as ** *p* < 0.01, *** *p* < 0.001 in comparison with the reference groups treated with ascorbic acid and β-glycerophosphate, as indicated.

In Figure 2, the results illustrate the impact of utilizing steamed fruit extract on the ALP activity of MC3T3-E1 cells. ALP is a key early marker of osteoblast differentiation and bone formation, often used to evaluate the osteogenic potential of various treatments [21]. ALP activity was assessed under non-cytotoxic conditions and expressed as a percentage relative to the untreated control group. The data revealed an increase in ALP activity when the cells were treated with steamed SH extracts. Specifically, ST-C led to a 6% increase in ALP activity at 25 µg/mL, which significantly rose to 26% at 100 µg/mL. Notably, ST-1 exhibited a remarkable (*p* < 0.001) 52% boost in ALP activity at 100 µg/mL when compared to ST-C. Furthermore, ST-2 yielded a 14% increase in ALP activity at 25 µg/mL, escalating to 60% at 100 µg/mL. Additionally, ST-3 resulted in a 36% rise in ALP activity at 100 µg/mL. These findings suggest that steamed SHF enhances ALP activity, which may support osteoblast differentiation and promote bone tissue formation, offering potential benefits in the context of osteoporosis.

### 2.5. Effect of Steaming of SHF on Mineralization and Calcium Deposition

**Figure 3:**
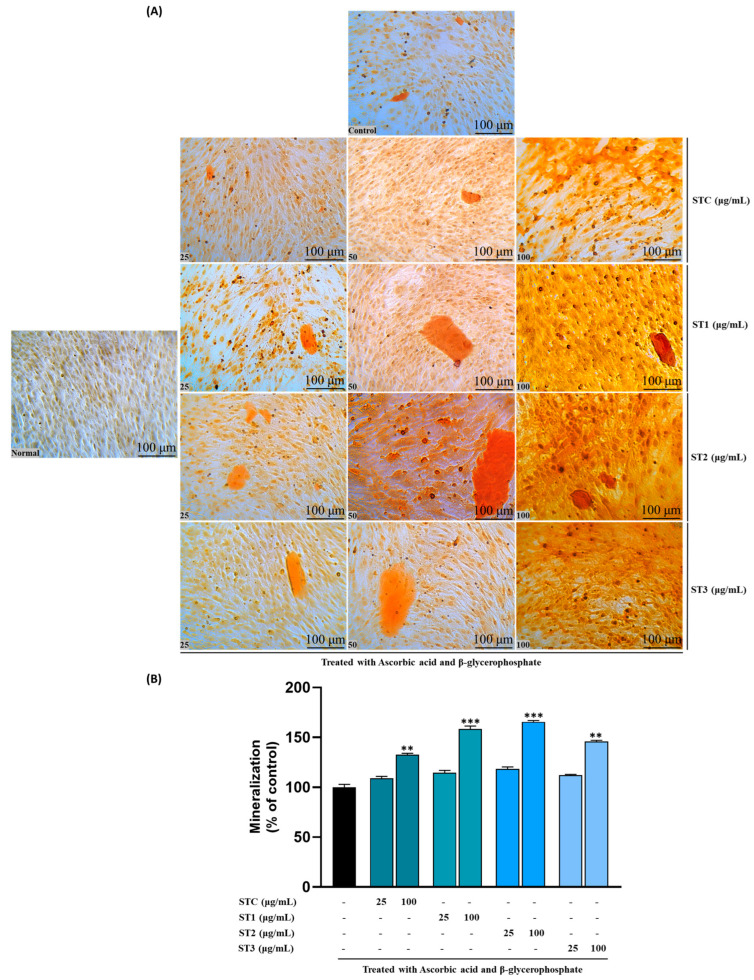
Effects of SHF extracts on mineralization in MC3T3-E1 cells. Cells were subjected to treatment with or without differentiation medium containing extracts at concentrations ranging from 25 to 100 μg/mL for a duration of 12 days. (**A**) Alizarin red staining was conducted, and the results were visualized through microscopy at ×100 magnification. (**B**) Mineralization (%) was quantified by measuring absorbance at 562 nm. The presented data represent the mean ± standard deviation from three independent experiments. Statistical analysis indicated significant differences, represented as ** *p* < 0.01, *** *p* < 0.001 in comparison with the respective ascorbic acid and β-glycerophosphate-treated group.

Mineralization is a crucial late-stage event in osteogenic differentiation, characterized by the deposition of calcium phosphate crystals into the extracellular matrix, which contributes to bone strength and integrity [22]. Our research outcomes revealed that the application of steamed SHF extract led to a significant increase in calcified extracellular matrix after 24 days when compared to the control group. Following ARS staining, the nodules of mineralized calcium deposition displayed a striking orange-red color. Specifically, the mineralization process exhibited substantial improvement, ranging from 106.1 ± 1.7% to 120.6 ± 1.36% with ST-C treatment at concentrations ranging from 25 to 100 μg/mL. Similarly, treatment with ST-1 resulted in enhanced mineralization, with values ranging from 108.5 ± 2.2% to 131.3 ± 3.0% at concentrations of 25 to 100 μg/mL. Likewise, ST-2 treatment at concentrations of 25 to 100 μg/mL increased the mineralization area from 111.4 ± 1.8% to 142.5 ± 1.3%, and ST-3 demonstrated an increase in mineralization ranging from 109.1 ± 0.89% to 126.9 ± 1.2% (Figure 3A,B). These findings reveal that steamed SHF extract significantly enhanced osteogenic mineralization in a dose-dependent manner.

### 2.6. Effect of Steaming of SHF on Collagen Content

**Figure 4:**
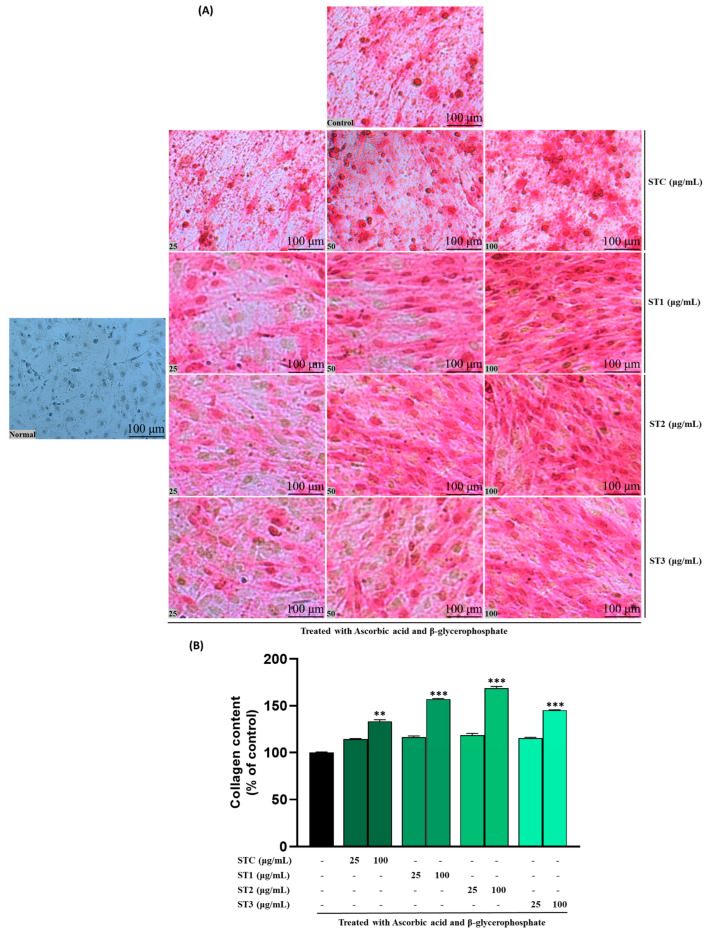
Effects of SHF extracts on collagen content in MC3T3-E1 cells. (**A**) Picro-Sirius red staining was carried out, and the outcomes were visualized through microscopy at ×100 magnification. (**B**) The collagen content was determined by measuring absorbance at 550 nm. The presented data represent the mean ± standard deviation from three independent experiments. Statistical analysis indicated significant differences, represented as ** *p* < 0.01, *** *p* < 0.001 in comparison with the respective ascorbic acid and β-glycerophosphate-treated group.

Collagen, particularly type I collagen, is a key structural protein in the bone extracellular matrix and plays a vital role in providing a scaffold for mineral deposition during osteogenesis [23]. Our findings indicate a positive influence of steam-treated SH on collagen formation, as illustrated in Figure 4A. The staining intensity of MC3T3-E1 cells exhibits a discernible dose-dependent trend. Concurrently, the quantitative outcomes presented in Figure 4B demonstrate notable dose-dependent variations among ST-C and the ST-1, ST-2, and ST-3 groups.

On day 14, a discernible increase of approximately 32% (in the ST-1 group), 40% (in the ST-2 group), and 25% (in the ST-3 group) was quantified across the concentration range of 0 to 100 μg/mL. These results highlight the positive impact of steam treatment on collagen synthesis and suggest a dose-dependent enhancement in the observed effects. In summary, steam-treated SH significantly promoted collagen synthesis in a concentration-dependent manner.

### 2.7. The Effect of Steaming of SHF on TRAP in RAW 264.7 Cells

Tartrate-resistant acid phosphatase (TRAP) is a well-established marker enzyme for osteoclast differentiation and activity, widely used to evaluate osteoclastogenesis and bone resorption processes. Osteoclasts are multinucleated cells responsible for bone resorption, and their overactivation is closely associated with pathological bone loss in conditions such as osteoporosis and arthritis [24]. We conducted further investigations to assess the impact of varying concentrations of steamed SH (ranging from 31.25 to 500 μg/mL) on osteoclast cell survival, osteoclastogenesis, and resorption activity. Following a 24 h treatment in RAW 264.7 cells, none of the tested dosages showed any detrimental effects. However, after 48 h, cell viability began to significantly decrease, particularly beyond the 125 μg/mL concentration (Figure S4A–C). Consequently, we selected 100 μg/mL as the highest concentration for subsequent experiments.

**Figure 5:**
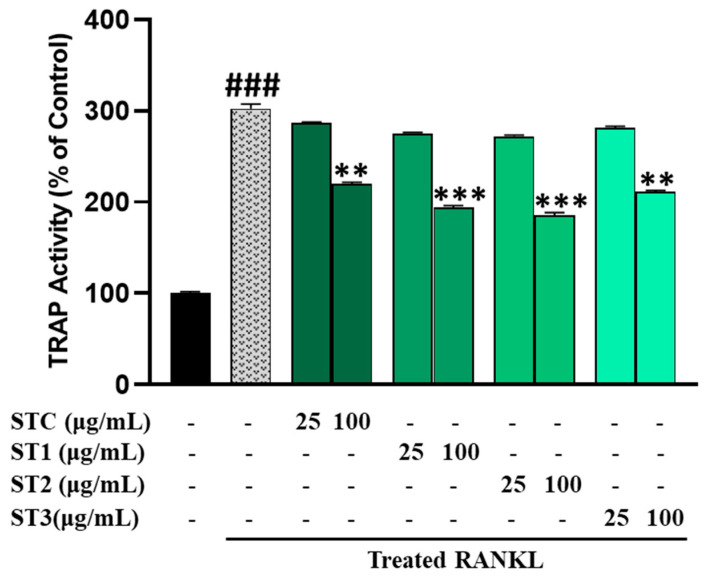
Osteoclast differentiation in RAW 264.7 cells was initiated by RANKL stimulation in the presence of SHF (25 to 100 µg/mL). After five days, TRAP (tartrate-resistant acid phosphatase) activity was quantified using the TRAP solution assay. The presented data represent the mean ± standard deviation from three independent experiments. Statistical analysis disclosed significant differences, denoted as ** *p* < 0.01, *** *p* < 0.001 compared to the specified RANKL-treated group and ### *p* < 0.001 compared to the control group.

**Figure 6:**
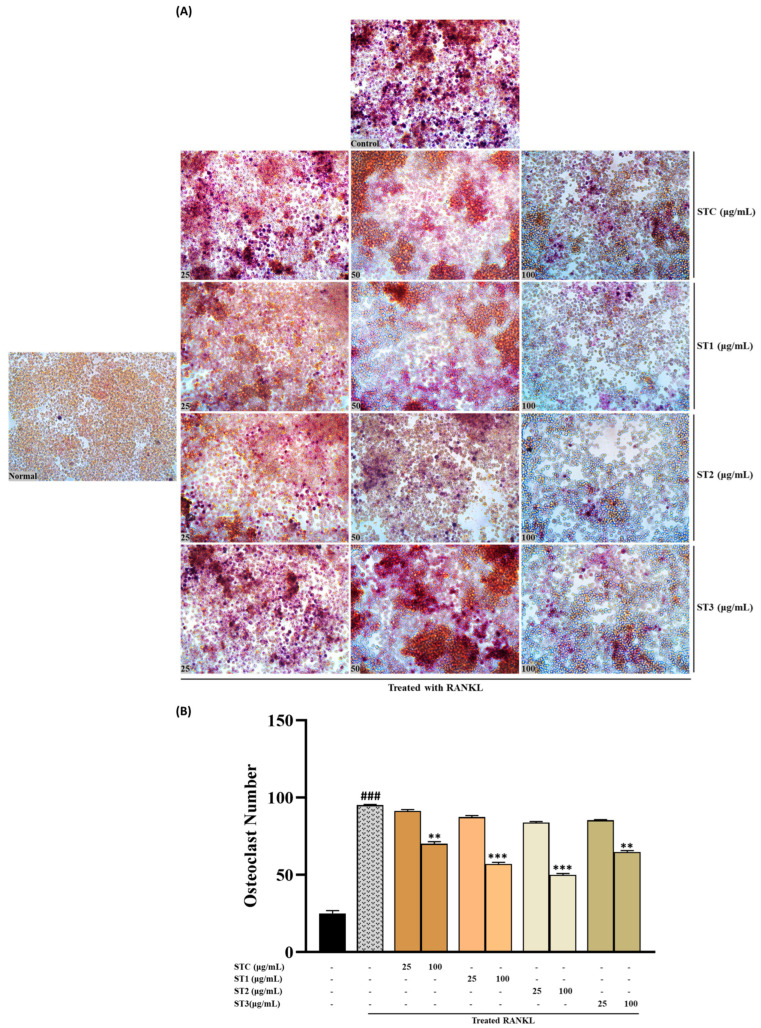
(**A**) Osteoclast differentiation induced by RANKL and influenced by SHF concentrations (25 to 100 μg/mL) over a 7-day period. (**A**) Osteoclast formation: Multinucleated osteoclast-like cells were observed at 100× magnification through light microphotography. Scale bars, 100 μm. (**B**) Enumeration of TRAP-positive multinucleated cells as indicative of osteoclasts. Statistical analysis indicated significant differences, represented as ** *p* < 0.01, *** *p* < 0.001 compared to the specified RANKL-treated group and ### *p* < 0.001 compared to the control group.

The results pertaining to the influence on TRAP activity, as measured in RAW 264.7 cells, are illustrated in Figure 5. In the presence of RANKL, RAW 264.7 cells generated multinucleated osteoclast-like (OCL) cells that exhibited TRAP positivity. Notably, the number of multinucleated cells showing TRAP positivity declined when exposed to steamed SHF extracts at concentrations of 25 and 100 μg/mL (Figure 6A).

It is worth mentioning that treatments with ST-1, ST-2, and ST-3 exhibited distinct morphological changes compared to control OCL cells, with reduced size and fewer nuclei. Specifically, ST-1 and ST-2 treatment significantly decreased the number of OCLs compared to RANKL-treated cells (Figure 6B). Similarly, steamed SHF extracts significantly reduced TRAP activity in a dose-dependent manner, with TRAP activity expressed as a relative value using the untreated group as a reference set at 100%. As depicted in Figure 5, even non-steamed fruits displayed a notable inhibitory effect on TRAP activity, amounting to approximately 219.7% (*p* < 0.05). However, the inhibition of TRAP activity was more pronounced in steamed fruits, with a notable increase in inhibitory activity as the number of steaming cycles increased. The inhibitory effect was notably higher when the fruit was steamed twice (*p* < 0.001). However, the inhibitory activity of fruits steamed three times on TRAP showed a slight reduction.

### 2.8. The Effect of Steaming of SHF on the Expression of Genes Related to the Differentiation and Proliferation of Osteoblasts and Osteoclasts

**Figure 7:**
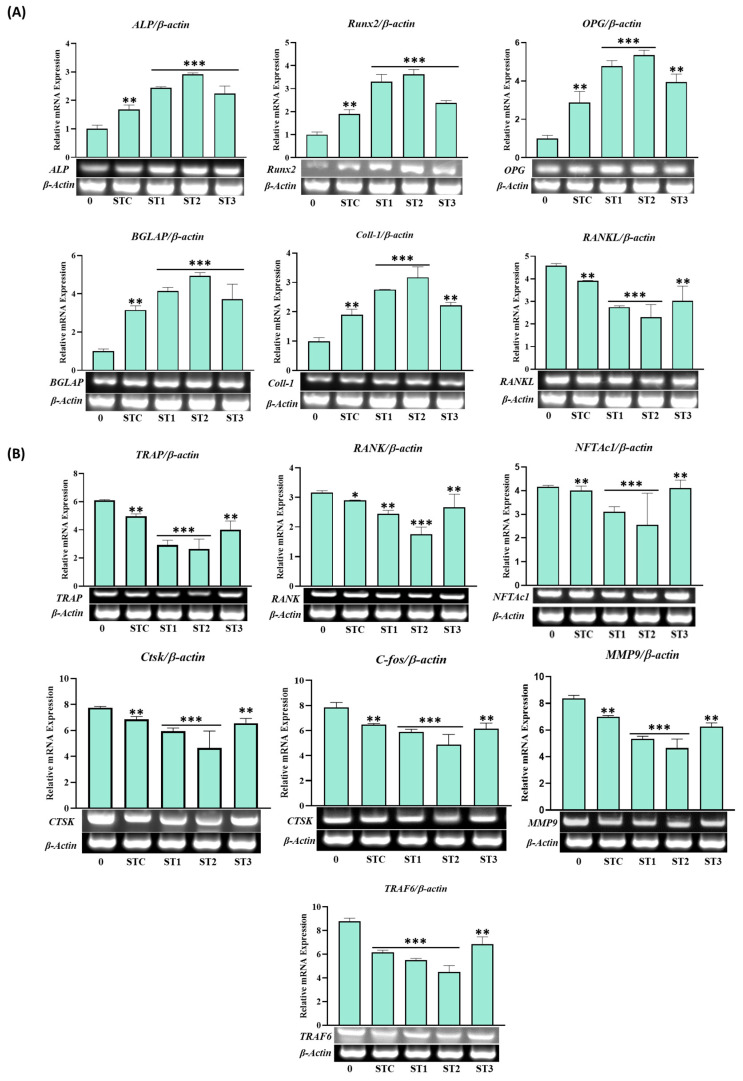
Effects of SHF extracts on osteoblast differentiation of MC3T3-E1 cells. Cells were treated with or without differentiation medium containing the extracts for a duration of 14 days. (**A**) Analysis of the relative mRNA expression levels of osteoblast markers (*Alp*, *Runx2*, *Col1-a1*, *BGLAP*, *OPG*, and *RANKL*) using real-time PCR. (**B**) Examination of the mRNA expression of transcriptional factors associated with osteoclast differentiation. RAW 264.7 cells were exposed to RANKL and SHF extracts for 7 days, and the expression of osteoclast differentiation marker genes (*TRAP*, *RANK*, *TRAF6*, *MMP9*, *c-Fos*, *CtsK*, *NFTAc1*) was measured using real-time PCR. Statistical analysis revealed significant differences, denoted as * *p* < 0.05, ** *p* < 0.01, *** *p* < 0.001 compared to the corresponding ascorbic acid and β-glycerophosphate-treated group and the RANKL-treated group, respectively.

To substantiate the promotion of osteogenic differentiation by steamed SH, we conducted an examination of osteogenesis-related markers through RT-PCR under 100 μg/mL concentrations of ST-C, ST-1, ST-2, and ST-3 after a 12-day osteogenic induction. The mRNA expression of Runt-related transcription factor 2 (Runx2) exhibited upregulation by 1.50 ± 0.18 (ST-C), 2.30 ± 0.31 (ST-1), 2.62 ± 0.20 (ST-2), and 1.98 ± 0.09 (ST-3) fold. Additionally, ALP, osteoprotegerin (OPG), type I collagen (Col1-a1), and Bone Gamma-Carboxyglutamate Protein (BGLAP) mRNA expression levels were assessed by RT-PCR (refer to Figure 7). Notably, the ST-C, ST-1, ST-2, and ST-3 treatment groups exhibited significant upregulation of *OPG*, *ALP*, *BGLAP*, and *Col1-a1* mRNA expression, with ST-1 and ST-2 demonstrating particularly noteworthy increases. Overall, the osteogenesis-related gene expression in the steamed ST-1 and ST-2 groups was approximately 30–60% higher than that in the ST-C treatment group. Furthermore, the expression of receptor activator of nuclear factor kappa beta (RANKL) exhibited a moderate decrease with ST-1, ST-2, and ST-3 treatments (Figure 7A).

To delve into the molecular mechanisms of steamed SH in osteoclast differentiation, we examined the expression of TRAF6, revealing that ST-1, ST-2, and ST-3 inhibited TRAF6 expression compared to ST-C. Subsequently, we investigated the mRNA expression of NFATc1 and c-Fos, key regulators of osteoclast differentiation. ST-1 and ST-2 significantly inhibited the mRNA expression of NFATc1 (*p*  <  0.001) and c-Fos (*p*  <  0.001) compared to both ST-C and ST-3. Further confirmation of the inhibitory effect on osteoclast differentiation involved RT-PCR detection of *RANK*, *TRAP*, *CtsK*, and *MMP9* expression (Figure 7B). ST-1, ST-2, and ST-3 demonstrated significant decreases in the expression levels of osteoclast differentiation-related genes compared to ST-C (Figure 7B), indicating that the inhibitory impact of steamed SH on RANKL-mediated *NFATc1* expression is followed by the downregulation of osteoclastogenic marker genes.

### 2.9. Osteotropic Effect of 5-HMF, a Metabolite Increased in SHF by Steaming Processes

**Figure 8:**
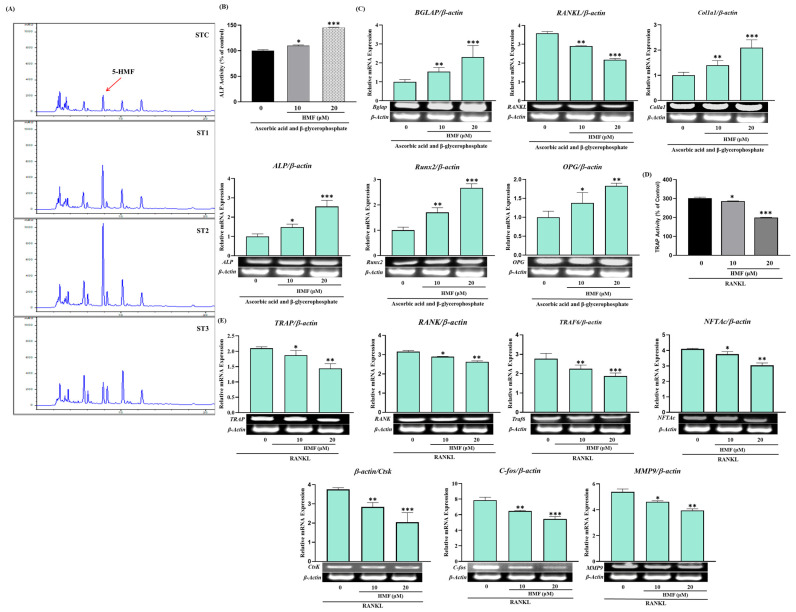
(**A**) Variation in HMF content due to steaming was assessed using high-performance liquid chromatography (HPLC). High-resolution chromatogram is provided in Figure S6. (**B**) Influence of HMF on ALP activity and (**C**) the corresponding gene expression. (**D**) Impact of HMF on TRAP activity and (**E**) the associated gene expression. Statistical analysis indicated noteworthy differences, represented as * *p* < 0.05, ** *p* < 0.01, *** *p* < 0.001 compared to the specified ascorbic acid and β-glycerophosphate-treated group and RANKL-treated group, respectively.

Notably, our study represents a novel contribution as we have successfully identified the presence of 5-HMF in the steamed fruit of SH. The verification of 5-HMF was achieved through the utilization of preparative High-Performance Liquid Chromatography (prep-HPLC) in conjunction with Nuclear Magnetic Resonance (NMR) spectroscopy. This analytical approach ensures the accuracy and reliability of our findings, further substantiating the observed increase in 5-HMF levels following steaming. In our investigation, the content of 5-HMF in untreated SHF was 217 µg/g as exists naturally, but it was significantly increased about two-fold by the once steamed process and showed a 4-fold increase by the twice steamed treatment. However, in the three times steamed SHF, it was reduced to almost the unsteamed level (Figure 8A). Figure 8B,C shows the effect of 5-HMF on ALP and genes related to the differentiation and proliferation of osteoblast cells. Interestingly 5-HMF markedly increased ALP activity in a dose-dependent manner, namely, the enzyme activity was increased 21% by 10 µg/mL 5-HMF and 43% by 20 µm/mL, respectively. 5-HMF also promoted the expression of the OPG, Rux2, and RANKL genes that play a key role in the differentiation and/or proliferation of osteoblast cells. 5-HMF strongly inhibited TRAP activity in RAW 264.7 ells and also suppressed the expression of its related genes such as RANK, TRAP, and TRAF6 (Figure 8D,E).

## 3. Discussion

In contemporary studies, there is growing interest in exploring natural products that can activate osteoblasts or hinder osteoclastogenesis. In this particular investigation, we examine the potential of steamed SHF as an alternative approach to combat osteoporosis, shedding light on its mechanism of action with a specific emphasis on in vitro osteogenesis.

Steaming SHF for up to three cycles increased browning in both the fruits and extract, likely due to the oxidation of diphenols and the Maillard reaction. Steaming twice boosted the extract yield by about 15% due to a looser texture. However, after three steam cycles, while browning intensified, the extract yield decreased compared to unsteamed fruits. This reduction is attributed to an increase in adhesive micronized particles, which, despite enhancing surface area, may have affected solubility. CGA is a representative phytochemical present in the fruits as it is a metabolite with various physiological activities, such as strong antioxidant activity, as well as bone formation-promoting and bone resorption-inhibiting activities [25]. Three CGA isomers—CGA, neo-CGA, and crypto-CGA—were found in similar amounts in SHF. The content of CGA remained stable after two steaming cycles but decreased by 25% after three cycles. The ratio of CGA isomers remained consistent up to two treatments, indicating CGA’s stability under steaming at 100 °C for 2 h. An interesting finding in this study is the remarkable increase in the TPC and TFC of SHF to 1.7- and 7.7-fold, respectively, by steaming two times.

This study demonstrated that SHF extract (100 μg/mL) increased ALP activity in osteogenic cells (MC3T3-E1) by approximately 26%, without causing cytotoxicity. Steaming the fruit further enhanced ALP activity, with a 52% increase after one steaming and a 60% increase after two steamings, at the same concentration. To determine whether the rise in ALP activity was due to a specific activator in the extract or cellular gene expression, gene expression analysis was performed. The results confirmed that the increased ALP activity was linked to the promotion of gene expression, rather than the simple action of a component in the extract.

The mineralization of the extracellular matrix is a critical process in bone formation, closely linked to the development and differentiation of osteoblasts [26]. The results of calcium nodule formation measured by ARS staining showed more clearly that the steaming of SHF can increase bone density as shown in Figure 3A. As another marker for mineralization, the anabolic effects of the steamed fruits on the differentiation and mineralization of osteoblasts were measured by evaluation of the collagen production in MC3T3-E1 cells. As shown in Figure 3B, the result of collagen production is almost the same pattern as ALP activity and the formation of calcium nodules.

TRAP is a glycoprotein produced by activated dendritic cells, mature osteoclasts, and macrophages and is recognized not only as a histochemical marker for osteoclasts but also as a widely occurring molecule with functions in both the skeletal and immune systems [24]. SHF extract significantly inhibited the activity of TRAP, and the inhibitory activity of the enzyme was further increased in steamed fruits. We also confirmed that the inhibition of this enzyme’s activity corresponded to the gene expression level by measuring the mRNA level.

Runx2 is a vital transcriptional regulator of osteoblast differentiation and bone formation that critically regulates many of the genes that control the development of osteoblast differentiation, such as ALP, Col1-a1, and OCN [27]. Therefore, we evaluated the effect of SHF extract on the expression of *Runx2*, *ALP*, *OCN*, *RANKL*, and *Col1-a1* by measuring the mRNA levels in MC3T3-E1 cells. Our results clearly show that steamed SHF extract may stimulate osteoblast differentiation and mineralization by signaling through the Runx2 pathway.

**Figure 9:**
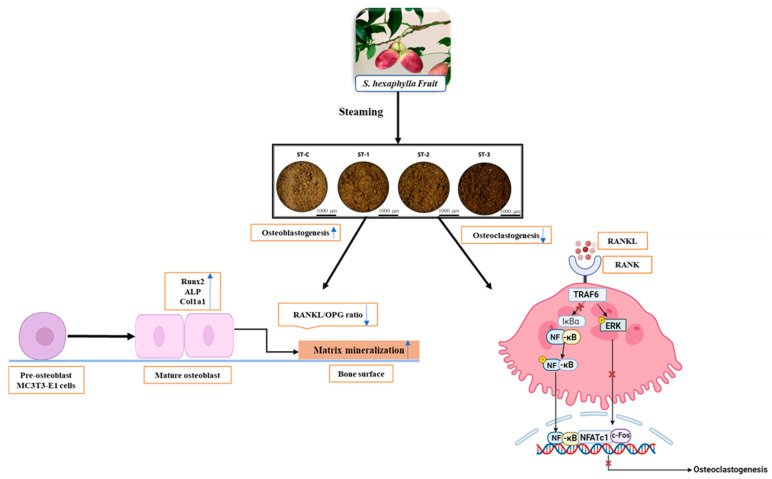
Visual summary of the methodology employed in this study.

In this study, SHF extract strongly inhibited the expression of key osteoclast-related genes induced by RANKL, particularly suppressing c-Fos and TRAF6. This suggests that SHF extract interferes with the early stages of osteoclast differentiation, potentially preventing excessive osteoclast formation and bone loss. The findings demonstrate that SHF extract not only promotes osteoblast differentiation but also inhibits osteoclast formation, highlighting its strong anti-osteoclastogenic activity (Figure 9). This dual effect may be attributed to specific components in SHF that target these pathways.

5-HMF is widely available in different food products that are thermally processed and it serves as a well-established indicator of nonenzymic browning, frequently employed to assess deteriorative changes resulting from heating and/or prolonged storage of food products [28]. It exhibits biphasic effects, that is, positive and negative activities in biological systems. Namely, 5-HMF has anti-cancer, antioxidant, anti-proliferative, anti-lesion, anti-inflammatory, anti-allergic, and anti-apoptotic activities [29].

In our studies, following one and two rounds of steaming, there was a substantial increase in HMF levels in SHF; however, a diminishing trend was observed after steaming three times. Therefore, the simple steaming of SHF can increase the physiological activity against osteoporosis, and since the fruit is high in sugar, it is also beneficial for long-term storage.

In summary, steaming SHF increases osteogenic activity, and the 5-HMF produced during steaming appears to contribute significantly. It seems that some components, including 5-HMF, present in SHF extract promote bone formation and inhibit bone resorption by regulating the expression of genes involved in the RANKL-RANK-OPG signaling pathway.

## 4. Materials and Methods

### 4.1. SHF Preparation and Steaming

The SHF used in this study was harvested in late November 2022 from a farm by Jeongnamjin *Stauntonia hexaphylla* Agricultural Cooperative Association located in Jangheung-gun Jeollanam-do, Republic of Korea (34°45′36.6″ N 126°53′55.5″ E), and authenticated by Professor Chulyung Choi of Jeonnam Institute of Natural Resources Research, Jangheung-gun Jeollanam-do, Republic of Korea. After harvesting, the sample was stored at −80 °C for two years until use.

Following authentication, the harvested SHF was immediately transported from the field. The samples were washed with tap water, cut into approximately 1.0 cm thick pieces, and dried at 45–50 °C for 24 h. Subsequently, 0.5 kg of the dried SHF was placed in a steamer (Cuckoo M-OC9600, Yangsan, Gyeongsangnam-do, Republic of Korea) and steamed for 2 h. During steaming, the internal temperature and pressure of the steamer were maintained at 110–114 °C and 70–80 kPa, respectively.

The dried steamed fruits and the unsteamed control were extracted separately with purified water at a 1:10 (*w*/*v*) ratio at 90 ± 3 °C for 6 h (Table S1). This extraction was performed twice, and the resulting extracts were combined and filtered. The combined extract was concentrated using a rotary evaporator, and the resulting concentrate was dissolved in distilled water for analysis.

### 4.2. Isolation of 5-HMF and Identification

The isolation of an unknown peak that highly increased from steamed SHF was carried out by a Waters prep-HPLC system (Milford, MA, USA) equipped with a Waters 1525 binary pump (manual injection valve) and 2489 UV/Visible detector. First, the extract of steamed SHF was placed in a separating funnel with ethyl acetate at a ratio of 1:1 (*v*/*v*) to carry out separation by solvent partition. Afterwards, the ethyl acetate layer was evaporated using a rotary evaporator. After evaporation, HPLC grade methanol was added to the dried extract, which was then subjected to prep-HPLC to separate the unknown peak. A Waters µBondapak^TM^ C18 Prep column (19 × 300 mm, 10 µm, Milford, MA, USA) was used as the stationary phase. The mobile phase was composed of 0.1% acetic acid aqueous solvent (A) and methanol (B). The flow rate was 12.28 mL/min, and the elution composition was initially 98% A and 2% B. The gradient elution composition was as follows: (0–20 min, 2% B; 20–30 min, 2–5% B, 30–40 min, 5% B; 40–50 min, 5–10% B; 50–51 min, 10–70% B; 51–60 min, 70% B). The injection volume was 1 mL, and the wavelength of the UV/VIS detector was 260 nm (Table S2). The collection of the unknown peak was determined by retention time. Initially, the eluting solvent was manually collected in a flask over a period of 3 to 10 min. The collected sample was subsequently evaporated and reconstituted in methanol for a second separation. During this step, the sample was separated in 1 min intervals from 6.5 to 10 min. The fractions obtained between 8 and 10 min were combined and evaporated, as this interval contained the peak of interest. Finally, it was weighed, and 18 mg of sample was subjected to NMR analysis. The determination of the molecular structure of this metabolite was performed by the Molecular Structure Determination Research Institute (MSDRI) of Kyung Hee University, Republic of Korea.

### 4.3. HPLC Analysis

The quantification of chlorogenic acids (CGAs) was carried out according to a protocol published elsewhere [30]. For the CGA, neo-CGA, and crypto-CGA contents, an HPLC system (Agilent Infinity 1260, Santa Clara, CA 95051, USA) was employed. The system comprised an Agilent 1260 Infinity Quaternary Pump (G1311B), Agilent 1260 Infinity Standard Auto Sampler (G1329B), Agilent 1260 Infinity Column Thermostat Compartment (G1316A), Agilent 1260 Infinity Variable Wavelength Detector (G1314F), and ZORBAX Eclipse Plus C18 (4.6 mm × 10 mm, 5 μm, Santa Clara, CA, USA), which served as the stationary phase. HPLC analysis was performed under the following conditions: an injection volume of 5 μL, a column temperature of 35 °C, a flow rate of 1 mL/min, and a detection wavelength of 260 nm. For the quantification of SHF metabolites, the SHF extract was first dried under reduced pressure. The dried extract was then reconstituted to a concentration of 1 mL per 1 g of the dried sample. Prior to analysis, the extract was diluted to one-fifth of its original concentration and subsequently injected into the HPLC system (Table S1 and Figure S5). The mobile phase consisted of solvent A (0.4% phosphoric acid) and solvent B (acetonitrile), with an initial elution composition of 95% A and 5% B. A gradient elution was performed as follows: (0–10 min, 5–9% B; 10–30 min, 9–9% B; 30–60 min, 9–30% B; 60–62 min, 30–50% B). Standards for CGA, neo-CGA, crypto-CGA, and 5-HMF were sourced from Sigma-Aldrich (St. Louis, MO, USA) and Wuhan ChemFaces Biochemical Co., Ltd. (Wuhan, China) with a purity of greater than 98%.

### 4.4. Phytochemical Analysis

#### 4.4.1. Determination of Polyphenol Content

The TPC was analyzed following the previous study with slight modification [31]. Briefly, 30 μL of the extraction solution was mixed with 150 μL of a 10% solution of 2 N Folin–Ciocalteu reagent. After vigorous shaking, the mixture stood at room temperature for 5 min, and then 160 μL of a 7.5% (*w*/*v*) sodium carbonate solution was added. The mixture was rested for 30 min in the dark, and its absorbance was measured at 715 nm using a spectrophotometer. A calibration curve using gallic acid as the reference standard allowed the computation of the TPC, expressed as μg gallic acid equivalent per milligram (GAE/mg) of the extract.

#### 4.4.2. Determination of Flavonoids

For TFC quantification, the extract (30 μL) was combined with distilled water (110 μL). Subsequently, 5% sodium nitrite (8 μL) was added, followed by the introduction of 10% aluminum chloride (8 μL) after 5 min. Next, the mixtures were allowed to stand for 6 min. Then, 1 M sodium hydroxide (50 μL) and distilled water (70 μL) were added, and the absorbance was evaluated at 510 nm. A calibration curve using rutin as the standard reference material enabled the calculation of the TFC, expressed as μg rutin equivalent (RE/mg) of the extract [31].

#### 4.4.3. Measurement of Free Radical Scavenging Ability

The assessment of the free radical scavenging capability was performed using the 2,2-diphenyl-1-picrylhydrazyl (DPPH) method. Specifically, each sample was appropriately diluted, and 180 μL of a 0.2 mM DPPH solution was combined with 20 μL of the sample within a 96-well plate. Following a 30 min incubation period in the absence of light, the absorbance was determined at 520 nm employing an enzyme-linked immunosorbent assay [32] reader (Bio-Tek Instruments, Inc., Vinooski, VT, USA). The radical scavenging capacity was quantified and expressed as μg GAE/g dry weight.

### 4.5. Pharmacological Efficacy Analysis

#### 4.5.1. Cell Viability Assay

MC3T3-E1 pre-osteoblast and RAW 264.7 macrophage cell lines were plated in 96-well culture plates at 1 × 10^4^ cells per well. After an overnight incubation period, the cells were subjected to treatment with several doses (between 31.25 µg/mL and 500 µg/mL) of steamed SH extract for a duration of 24 to 48 h. Cell viability was assessed using 3-(4,5-dimethylthiazol-2-yl)-2,5-diphenyltetrazolium bromide (MTT) assay solutions after 2 days of incubation.

To conduct the MTT assay, 20 μL of MTT solution (5 mg/mL) was added per well, and the plates were left to incubate for 3 to 4 h. Following this incubation period, the MTT solutions were aspirated, and 100 µL of dimethyl sulfoxide (DMSO) was added to dissolve the formazan product. Subsequently, the absorbance was measured at 570 nm using a microplate reader (BioTek Instruments, Inc., Winooski, VT, USA). The relative cell viability was expressed as a percentage in comparison to the untreated control cells.

#### 4.5.2. Osteoblast Cell Culture and Differentiation

A pre-osteoblast cell line, MC3T3-E1 (RCB1126, from C57BL/6 mouse calvaria), was obtained from the RIKEN Cell Bank (Tsukuba, Ibaraki, Japan) and was maintained in α-MEM (Gibco-BRL, Grand Island, NY, USA). The media were supplemented with 10% heat-inactivated fetal bovine serum (Gibco-BRL, Gaithersburg, MD, USA) and 1% penicillin–streptomycin (Gibco-BRL, Gaithersburg, MD, USA), which was called complete medium (CM). Cells were then allowed to adhere at 37 °C in a humidified 5% CO_2_ incubator (Thermo Electron Corporation, Waltham, MA, USA). After reaching 80% to 90% confluence, the cells were seeded in a 12-well plate at a density of 5 × 10^3^. Cell differentiation was induced by adding 10 mM glycerophosphate and 50 µg/mL ascorbic acid in CM, which was called differentiation medium (DM), for an additional 6 to 24 days.

#### 4.5.3. Alkaline Phosphatase (ALP) Activity Assay

The ALP activity of differentiated osteoblasts was measured using an ALP assay kit from Sigma Chemical (St. Louis, MO, USA) following the manufacturer’s instructions. Briefly, MC3T3-E1 cells were differentiated in a 12-well plate using 10 mM glycerophosphate and 50 µg/mL ascorbic acid in CM with or without SH extracts. After 12 days, the cell monolayers were lysed with 0.1% Triton X-100/PBS followed by a PBS wash for three times. After that, the cell lysates were centrifuged at 12,000 rpm for 5 min at 4 °C, and the supernatant was collected in 1.5 mL tubes for ALP activity measurement. The cell supernatant and *p*-nitrophenyl phosphate (pNPP) substrate were added in a 96-well plate and incubated for 10 min at 37 °C. The reaction was stopped using a stop solution after 10 min. Finally, the optical density (OD) values at 405 nm were recorded using an ELISA reader (Bio-Tek Instruments, Inc., Vinooski, VT, USA). Lastly, the ALP activity was normalized using the bicinchoninic acid (BCA) kit (Sigma Chemical, St. Louis, MO, USA).

#### 4.5.4. Alizarin Red Staining

MC3T3-E1 cells were differentiated in the presence or absence of SH extracts, as detailed in previous protocols [33]. After 24 days, the cells were first meticulously rinsed twice with PBS, preserved with para-formaldehyde (4%) for 20 min, and subsequently dyed with 2% Alizarin Red S dye solution (Sigma-Aldrich) adjusted to pH 4.2 at room temperature for 10 min. The extracellular matrix mineralization nodules were photographed using a digital camera attached to an inverted microscope.

Subsequently, to analyze the ARS staining, a resolving solution containing 10% acetic acid and 20% methanol was used to dissolve the cells. After drying, the resulting liquid was transferred to a 96-well plate 15 min later. The absorbance was measured at a wavelength of 450 nm using an ELISA reader (Epoch TM Microplate Spectrophotometer: BioTek Instruments Inc., Winooski, VT, USA). Each reaction was carried out in triplicate.

#### 4.5.5. Collagen Content

MC3T3-E1 cells were cultivated using the same protocols used for the ALP assays to measure the amounts of cellular collagen. After an overnight incubation period and confluence, cells were treated with DM (with or without SH extracts) to start the differentiation process for a further 12 days. Every two days, the cultural medium was refreshed.

After the 12-day treatment period, the collagen content was quantified using a Sirius Red-based colorimetric assay. In brief, the cells were rinsed twice with PBS and then exposed to Bouin’s solution for 1 h. After removing Bouin’s solution, the cells were rinsed by running tap water over the culture plates for 10 to 15 min. The plates were allowed to air-dry and then were stained with a reagent containing Sirius Red dye for 1 h with gentle agitation. After this staining time, 0.01 N HCl was used to wash the cells to remove any remaining dye. After dissolving the dyed material in 0.1 N NaOH, the absorbance of the resultant solutions was measured with a microplate reader at 550 nm.

#### 4.5.6. Osteoclast Cell Culture and Differentiation

The mouse macrophage-like RAW 264.7 cells were obtained from Ginseng Bank (Kyung Hee University, Republic of Korea). The cells were cultured in a CM that consists of 89.5% Dulbecco’s modified Eagle’s medium, 10% fetal bovine serum (FBS), and 100 U/mL penicillin–streptomycin and maintained in an incubator containing 5% CO_2_ at 37 °C. Cells were grown in 12-well plates (5 × 10^3^ cells/well) with or without 50 ng/mL of RANKL once they had reached 80% to 90% confluence. Cells were cultured for an additional 3–7 days, and multinucleated osteoblast cells were visible at day 7.

#### 4.5.7. TRAP Activity Assay

The RAW 264.7 cells were cultured and differentiated with 100 ng/mL RANKL in the presence or absence of SH extract for 5 to 7 days. After osteoblast cell differentiation, cell monolayers were washed with PBS and centrifuged at 12,000 rpm for 5 min, followed by lysing with 0.5% Triton X-100. The supernatant was used for the quantification of activity using the TRAP staining kit (Sigma Chemical, St. Louis, MO, USA) according to the manufacturer’s protocol.

#### 4.5.8. RNA Isolation and Real-Time Reverse Transcription-PCR (qRT-PCR) Analysis

Following the differentiation and treatment of MC3T3-E1 and RAW 264.7 cells in the presence or absence of SHF extracts, total RNA was extracted using TriZol LS reagents (Invitrogen, Carlsbad, CA, USA), adhering to the manufacturer’s instructions. Moreover, employing the RevertAid First Strand cDNA Synthesis Kit from Thermo Fisher Scientific, USA, we generated 20 μL of cDNA from 2.5 ng of RNA. This was achieved by incubating the mixture at 42 °C for 45 min, followed by a 70 °C incubation for 5 min, as per the manufacturer’s guidelines. The entire procedure was conducted within a PCR-clean environment.

Real-time reverse transcription-PCR (qRT-PCR) was carried out to gauge the gene expression using an Invitrogen SYBR Green qPCR Super Mix UDG kit and an R-Corbett Rotor-Gene Model 6000 (Mortlake, NSW, Australia). The relative expression of gene-specific products was assessed and normalized to the corresponding β-actin levels, applying the 2^−∆∆Ct^ method. These results were verified through three independent experiments. The list of primers can be found in Table S3.

#### 4.5.9. Statistical Analysis

The data are presented as the mean ± standard error (SE) derived from a minimum of three distinct and independent experimental replicates. Analysis of the data was performed using GraphPad Prism software 8.0.2 (GraphPad Software, La Jolla, CA, USA). The total variations between treated groups and untreated (control) groups were determined by Student’s t-test and two-way analysis of variance (ANOVA). Statistical significance was determined at a significance level of *p* < 0.05.

## 5. Conclusions

This study is the first to demonstrate in vitro the positive effects of SHF extract on osteoblastogenesis and osteoclastogenesis during osteoporosis. The steaming of SHF increases the yield of the extract without affecting the content of CGA, which is one of the main components. It also significantly increases the contents of phenolics including flavonoids and desirable Maillard reaction products, as well as antioxidant activity. More interestingly, this process significantly improved the bone formation-promoting and bone resorption-inhibiting activities at the cellular level. This study provided valuable information on the dynamic changes in phenolic profiles and the ability of SHF to affect bone cell differentiation after a steaming process, which could offer one possible strategy for improving the biochemical activity of SHF.

Despite the strengths of our study, several limitations should be addressed. First, our experiments utilized secondary cell lines such as MC3T3-E1 and RAW 264.7, which, while widely accepted for studying osteoblast and osteoclast differentiation, may not fully capture the complexity of the in vivo bone microenvironment. To better validate our findings, future studies should employ primary cell lines, such as bone marrow-derived macrophages, to more accurately model physiological conditions. Additionally, while we observed promising results related to osteogenic activity and inhibition of osteoclastogenesis, our study only provides a limited investigation into the specific signaling pathways involved. The precise mechanisms by which SHF extracts modulate key osteogenic and osteoclastogenic pathways, such as the Wnt, BMP, or RANKL pathways, need to be explored in greater detail. Further research is necessary to elucidate these signaling cascades and their downstream targets. Clinical trials are essential to establish the effective dose, safety, and therapeutic potential of SHF in humans. Addressing these limitations will provide a more comprehensive understanding of SHF’s role as a potential treatment or preventive measure for osteoporosis.

## Data Availability

The experimental data are included in the manuscript and are available from the corresponding author upon request.

## Supplementary Materials

The following supporting information can be downloaded at: https://www.mdpi.com/article/10.3390/ijms26178411/s1.

## Abbreviations

The following abbreviations are used in this manuscript:

5-HMF	5-Hydroxymethylfurfural
SHF	*Stauntonia hexaphylla* (Thunb.) Decne fruit
Prep-HPLC	Preparative HPLC
TPC	Total phenolic content
TFC	Total flavonoid content
RANKL	Receptor activator of nuclear factor kappa beta
CGAs	Chlorogenic acids
TRAP	Tartrate-resistant acid phosphatase
ALP	Alkaline phosphatase
ST-C	Unsteamed *S. hexaphylla* fruit extract
ST-1	Once steamed *S. hexaphylla* fruit extract
ST-2	Twice steamed *S. hexaphylla* fruit extract
ST-3	Three times steamed *S. hexaphylla* fruit extract
DM	Differentiation medium
ELISA	Enzyme-linked immunosorbent assay
pNPP	*p*-Nitrophenyl phosphate
OD	Optical density
BCA	Bicinchoninic acid
