# Supplementary material for: Steam-Processed Stauntonia hexaphylla (Thunb.) Decne Fruit Stimulates Osteoblast Differentiation in MC3T3-E1 Cells and Inhibits Osteoclastogenesis in RAW 264.7 Cells
Source: Int J Mol Sci. 2025 Aug 29;26(17):8411. doi: 10.3390/ijms26178411 (PMC12428769; doi:10.3390/ijms26178411)
Supplement: Supplementary file 1 [file ijms-26-08411-s001.zip › ijms-3576803-supplementary.pdf]

Steam-processed *Stauntonia hexaphylla* (Thunb.) Decne fruit stimulates osteoblast differentiation in MC3T3-E1 cells and inhibits osteoclastogenesis in RAW 264.7 cells

Samples	ST-C	ST-1	ST-2	ST-3
Fruits color	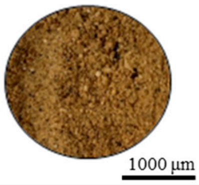	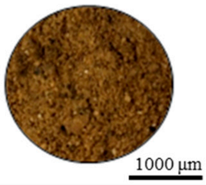	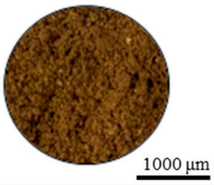	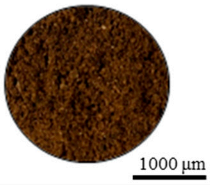
Yield (mg solid/g)	440	464	506	415

Figure S1. Alteration in the color and extract yield of SHF through the steaming process. ST-C: unsteamed *Stauntonia hexahylla* fruits; ST-1: *Stauntonia hexahylla* fruits were steamed once; ST-2: *Stauntonia hexahylla* fruits were steamed twice, ST-3: *Stauntonia hexahylla* fruits were steamed thrice.

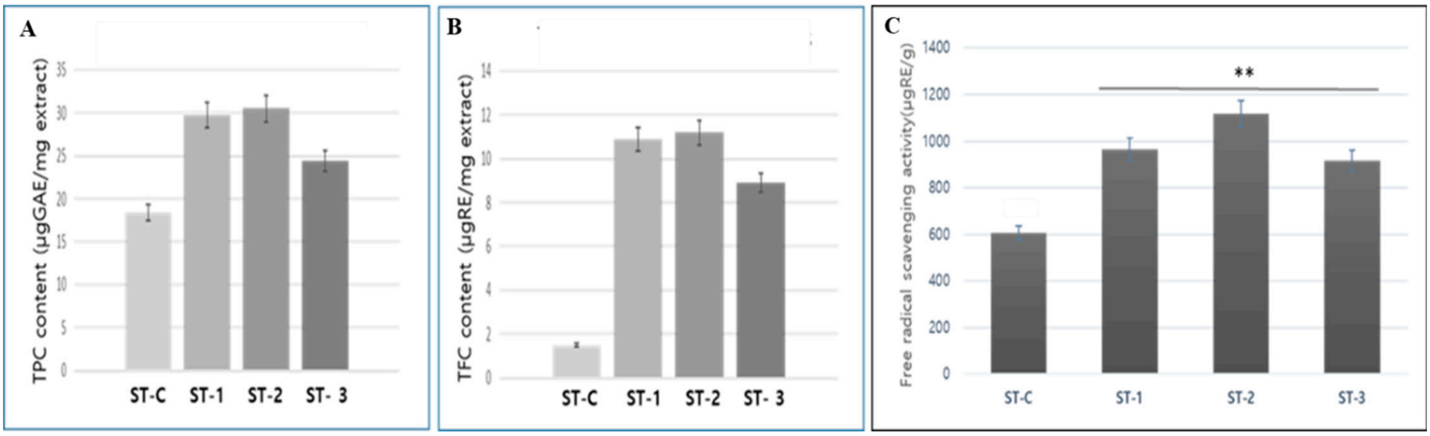

Figure S2. Changes in the contents of (A) total phenolic, (B) flavonoid metabolites, as well as the (C) antioxidant capacity of SHF, resulting from the steaming treatment. ST-C: unsteamed *Stauntonia hexahylla* fruits; ST-1: Stauntonia hexahylla fruits were steamed once; ST-2: *Stauntonia hexahylla* fruits were steamed twice, ST-3: *Stauntonia hexahylla* fruits were steamed thrice. Statistical analysis indicated significant differences, marked as **$p<0.01$, in comparison with the ST-C group.

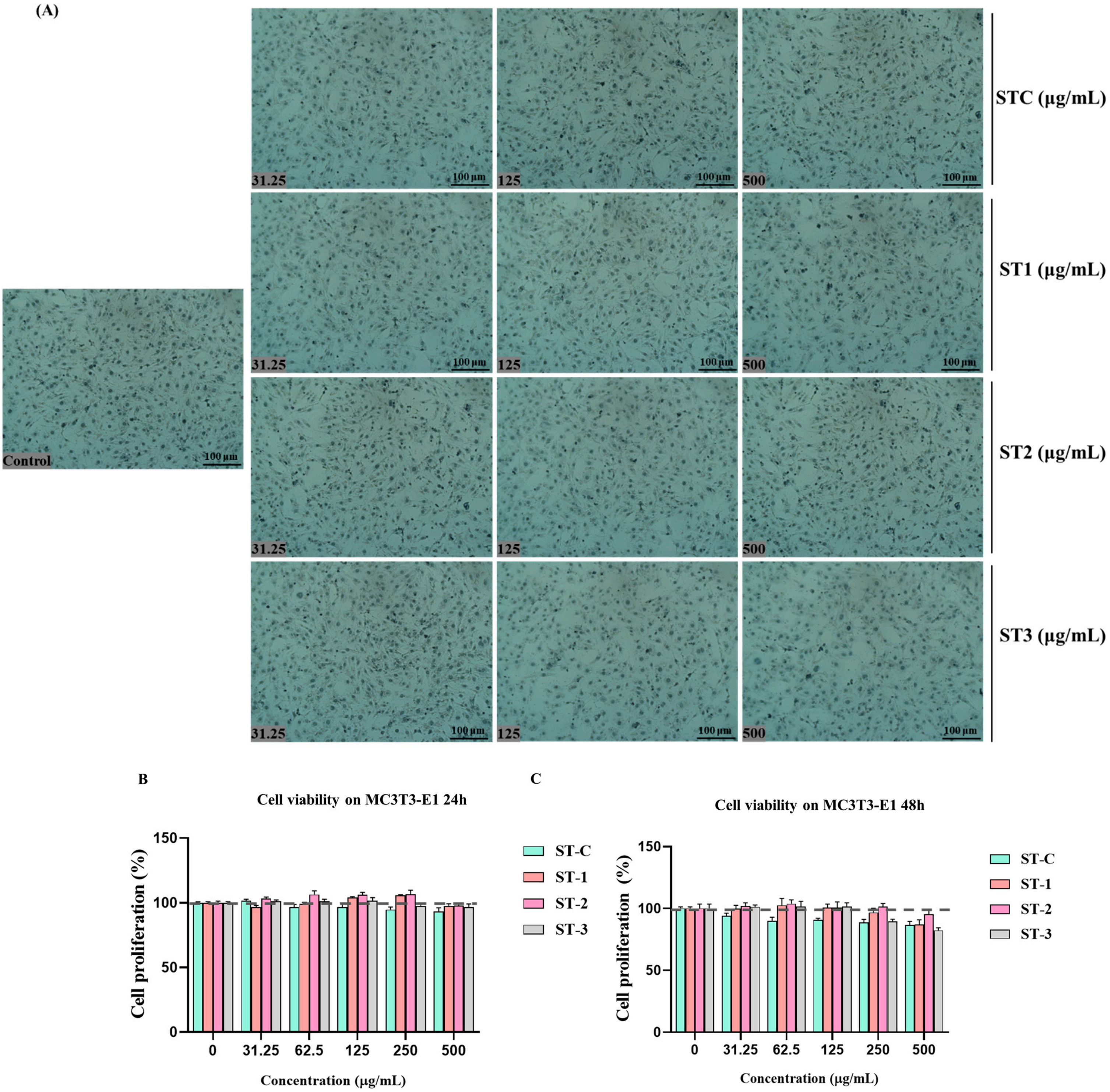

Figure S3. Visualization of cellular morphology through phase-contrast microscopy (×20 magnification) after the 2-day treatment with SHF. Scale bar = 100 µm. (A). (B) C Assessment of cell viability in MC3T3-E1 pre-osteoblast cells (1×104 cells/well) treated with varying concentrations of SHF (0–500 µg/ml) for 24h (C) 48h., using an MTT assay.

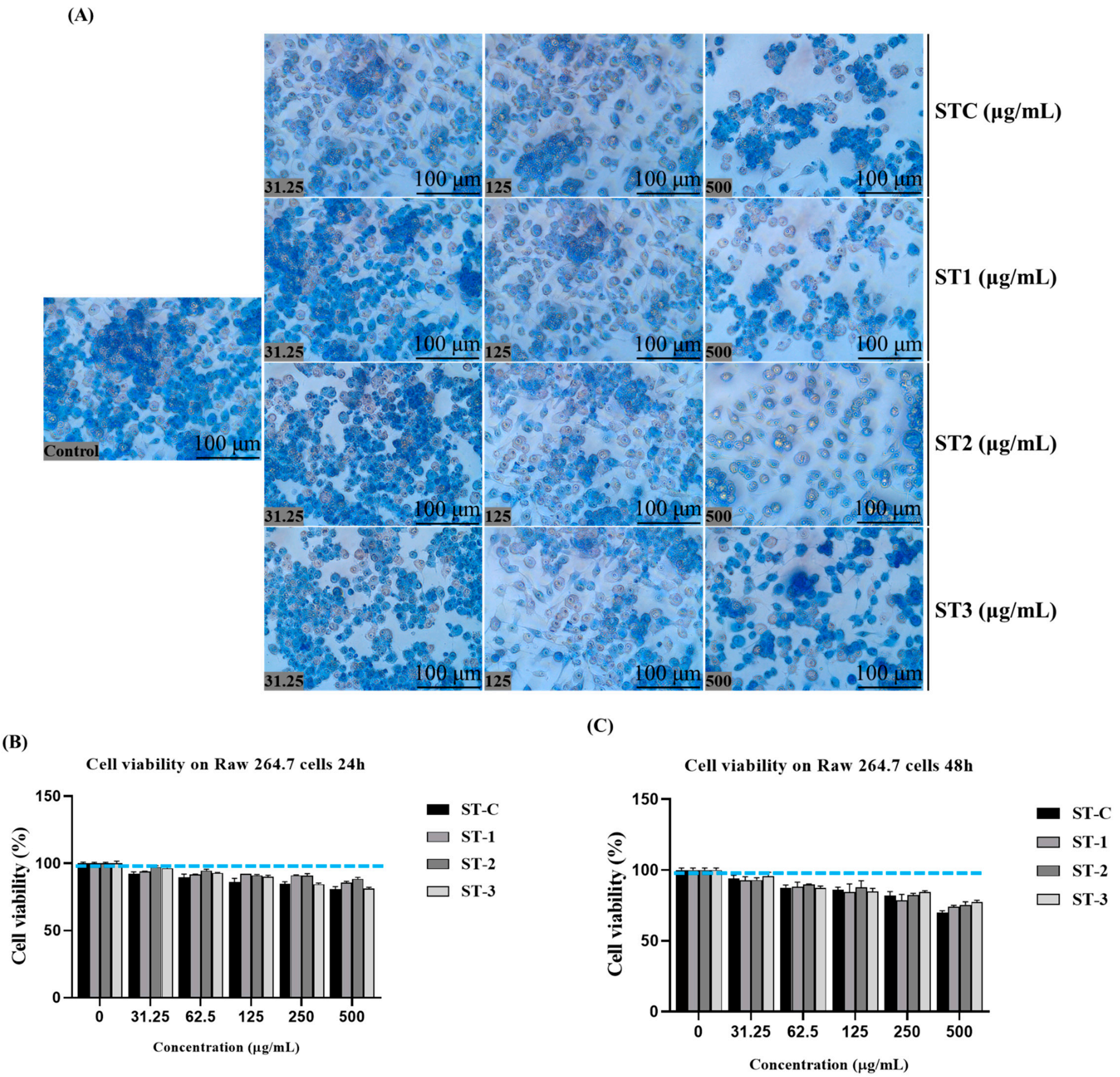

Figure S4. RAW 264.7 macrophage cells (1×10^4 cells/ml) were exposed to varying concentrations of SHF (0–500 µg/ml) for a duration of 2 days. (A) Cell viability was assessed through an MTT assay at 24h and 48h. (B) Cellular images were captured using phase-contrast microscopy (×20 magnification). Scale bar = 100 µm.

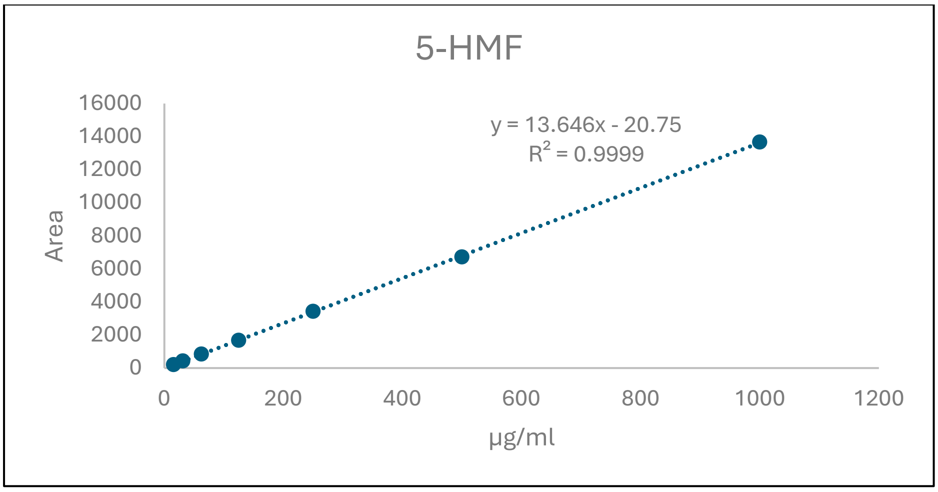

Figure S5: Standard calibration curve for 5-HMF.

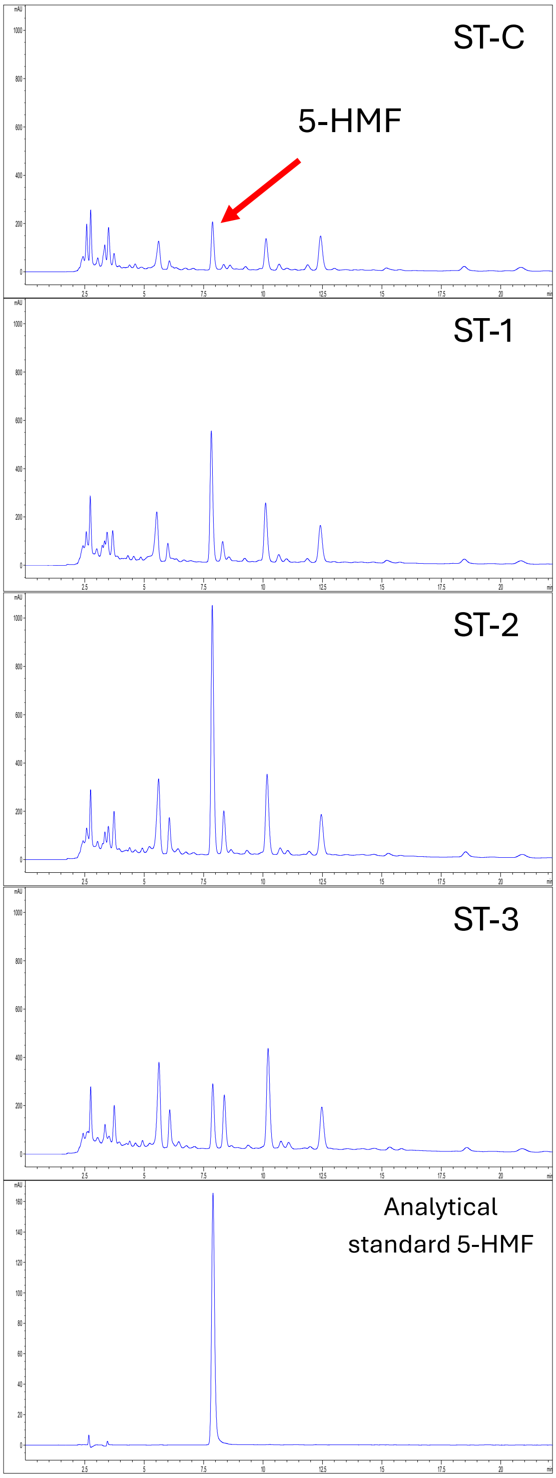

Figure S6: High-resolution chromatogram of HPLC.

Table S1: Effect of solvents, temperature, and extraction No. on SHF extraction yield.

No.	Solvents	Temperature	Solvent Volume (Ratio to raw materials)	No of extraction	Amount extracted (g, brix)	Yield (%)
1	100% water	80 ℃	10x	1	95.84g, 50brix	24.0
2		90 ℃	10x	1	101.16g, 51brix	25.8
3		100 ℃	10x	1	110.68g, 51brix	28.2
4	70% water + 30 ethanol	50 ℃	10x	1	90.68g, 51brix	23.1
5		60 ℃	10x	1	87.47g, 50brix	21.9
6		70 ℃	10x	1	100.13g, 51brix	25.5
7	50% water + 50 ethanol	50 ℃	10x	1	96.95g, 50brix	24.2
8		60 ℃	10x	1	83.07g, 51brix	21.2
9		70 ℃	10x	1	72.41g, 50brix	18.1
10	100% water	90 ℃	10x	1	101.16g, 51brix	25.8
11			10x	2	145.79g, 50brix	36.5
12			10x	3	147.47g, 50brix	36.9
13	70% water + 30 ethanol	70 ℃	10x	1	100.13g, 51brix	25.5
14			10x	2	129.97g, 50brix	32.5
15			10x	3	142.72g, 50brix	35.7
16	50% water + 50 ethanol	70 ℃	10x	1	72.41g, 50brix	18.1
17			10x	2	103.24g, 50brix	25.8
18			10x	3	126.42g, 50brix	31.6
19	100% water	90 ℃	10x	2	145.8g, 51brix	36.4
20			15x	2	146.7g, 51brix	36.7
21			20x	2	147.1g, 50brix	36.9

Table S2. Quantification of 5-HMF

Samples	Steaming (120min)	conc. (µg/ml)	content (µg/g)
ST-C	No steaming	48.09	217.3
ST-1	1x	130.98	589.0
ST-2	2x	226.02	1062.3
ST-3	3x	63.92	291.4

**Gene**	**Forward primer**	**Reverse primer**	**Reference**
ß-Actin	ATGAAGTGTGACGTTGACATCC	CCTAGAAGCATTTGCGGTGCACGATG	[1]
ALP	CGAGCAGGAACAGAAGTTTGC	TGGCCAAAAGGCAGTGAATAG	
Runx2	ATGGCCGGGAATGATGAGAA	TCTGTCTGTGCCTTCTTGGT	[2]
Col1-a1	GATGGATTCCAGTTCGAGTATG	GTTTGGGTTGCTTGTCTGTTTG	
OPG	ACAATGAACAAGTGGCTGTGCTG	CGGTTTCTGGGTCATAATGCAAG	[3]
RANKL	GCAGCATCGCTCTGTTCCTGTA	GCATGAGTCAGGTAGTGCTTCTGTG	[4]
BGLAP	GTGCAGACCTAGCAGACACCA	GTAGCGCCGGAGTCTATTCA	[5]
TRAP	CTGCTGGGCCTACAAATCAT	GGTAGTAAGGGCTGGGGAAG	[6]
RANK	AGAAGACGGTGCTGGAGTCT	TAGGAGCAGTGAACCAGTCG	
MMP9	GACGGCACGCCTTGGTGTAG	AGGAGCGGCCCTCAAAGATG	[7]

Ctsk	ATATGTGGGCCACCATGAAAGTT	TCGTTCCCCACAGGAATCTCT	
c-fos	GGGACAGCCTTTCCTACTACC	GATCTGCGCAAAAGTCCTGT	[8]
TRAF6	TGCGGGTCCAGCCAGTCGT	TTCCCGTAAAGCCATCAAGCAGA	[9]
NFATc1	GGTAACTCTGTCTTTCTAACCTTAAGCTC	GTGATGACCCCAGCATGCACCAGTCACAG	[10]

Table S3. Primer list.

1. Siraj, F.M.; SathishKumar, N.; Kim, Y.J.; Kim, S.Y.; Yang, D.C. Ginsenoside F2 possesses anti-obesity activity via binding with PPARγ and inhibiting adipocyte differentiation in the 3T3-L1 cell line. *Journal of enzyme inhibition and medicinal chemistry* **2015**, *30*, 9-14.
2. Siddiqi, M.H.; Siddiqi, M.Z.; Ahn, S.; Kim, Y.-J.; Yang, D.C. Ginsenoside Rh1 induces mouse osteoblast growth and differentiation through the bone morphogenetic protein 2/runt-related gene 2 signalling pathway. *Journal of Pharmacy and Pharmacology* **2014**, *66*, 1763-1773.
3. Zhao, J.-J.; Wu, Z.-F.; Wang, L.; Feng, D.-H.; Cheng, L. MicroRNA-145 mediates steroid-induced necrosis of the femoral head by targeting the OPG/RANK/RANKL signaling pathway. *PLoS One* **2016**, *11*, e0159805.
4. Zhou, L.-P.; Wong, K.-Y.; Yeung, H.-T.; Dong, X.-L.; Xiao, H.-H.; Gong, A.G.-W.; Tsim, K.W.-K.; Wong, M.-S. Bone protective effects of danggui buxue tang alone and in combination with tamoxifen or raloxifene in vivo and in vitro. *Frontiers in pharmacology* **2018**, *9*, 779.
5. Skafi, N.; Abdallah, D.; Soulage, C.; Reibel, S.; Vitale, N.; Hamade, E.; Faour, W.; Magne, D.; Badran, B.; Hussein, N. Phospholipase D: A new mediator during high phosphate-induced vascular calcification associated with chronic kidney disease. *Journal of cellular physiology* **2019**, *234*, 4825-4839.
6. Li, L.; Yang, M.; Shrestha, S.K.; Kim, H.; Gerwick, W.H.; Soh, Y. Kalkitoxin reduces osteoclast formation and resorption and protects against inflammatory bone loss. *International Journal of Molecular Sciences* **2021**, *22*, 2303.
7. Lee, Y.D.; Yoon, S.-H.; Ji, E.; Kim, H.-H. Caveolin-1 regulates osteoclast differentiation by suppressing cFms degradation. *Experimental & Molecular Medicine* **2015**, *47*, e192-e192.
8. Bougault, C.; Aubert-Foucher, E.; Paumier, A.; Perrier-Groult, E.; Huot, L.; Hot, D.; Duterque-Coquillaud, M.; Mallein-Gerin, F. Dynamic compression of chondrocyte-agarose constructs reveals new candidate mechanosensitive genes. *PloS one* **2012**, *7*, e36964.

9. Mao, X.; Pan, X.; Zhao, S.; Peng, X.; Cheng, T.; Zhang, X. Protection against titanium particle-induced inflammatory osteolysis by the proteasome inhibitor bortezomib in vivo. *Inflammation* **2012**, *35*, 1378-1391.
10. Hajavifard, N.; Matinhomaee, H.; Hosseini, S.A. Effect of Aerobic Training and Vitamin D Consumption on NFATc1 Gene Expression in Bone Tissue of Rats Exposed to H2O2. *Gene, Cell and Tissue* **2020**, *7*.
